# Sfcnn: a novel scoring function based on 3D convolutional neural network for accurate and stable protein–ligand affinity prediction

**DOI:** 10.1186/s12859-022-04762-3

**Published:** 2022-06-08

**Authors:** Yu Wang, Zhengxiao Wei, Lei Xi

**Affiliations:** 1 Chongqing Key Laboratory of Big Data for Bio Intelligence, Chongqing University of Posts and Tele-Communications, No. 2 Chongwen Road, Nan’an District, Chongqing, 400065 China; 2 Department of Clinical Laboratory, Public Health Clinical Center of Chengdu, Chengdu, 610095 China; 3 Hubei Provincial Key Laboratory of Occurrence and Intervention of Rheumatic Diseases, Hubei Minzu University, Enshi, 445000 China

**Keywords:** Scoring function, Protein–ligand binding affinity, Convolutional neural network, Sfcnn

## Abstract

**Background:**

Computer-aided drug design provides an effective method of identifying lead compounds. However, success rates are significantly bottlenecked by the lack of accurate and reliable scoring functions needed to evaluate binding affinities of protein–ligand complexes. Therefore, many scoring functions based on machine learning or deep learning have been developed to improve prediction accuracies in recent years. In this work, we proposed a novel featurization method, generating a new scoring function model based on 3D convolutional neural network.

**Results:**

This work showed the results from testing four architectures and three featurization methods, and outlined the development of a novel deep 3D convolutional neural network scoring function model. This model simplified feature engineering, and in combination with Grad-CAM made the intermediate layers of the neural network more interpretable. This model was evaluated and compared with other scoring functions on multiple independent datasets. The Pearson correlation coefficients between the predicted binding affinities by our model and the experimental data achieved 0.7928, 0.7946, 0.6758, and 0.6474 on CASF-2016 dataset, CASF-2013 dataset, CSAR_HiQ_NRC_set, and Astex_diverse_set, respectively. Overall, our model performed accurately and stably enough in the scoring power to predict the binding affinity of a protein–ligand complex.

**Conclusions:**

These results indicate our model is an excellent scoring function, and performs well in scoring power for accurately and stably predicting the protein–ligand affinity. Our model will contribute towards improving the success rate of virtual screening, thus will accelerate the development of potential drugs or novel biologically active lead compounds.

**Supplementary Information:**

The online version contains supplementary material available at 10.1186/s12859-022-04762-3.

## Background

Modern drug development has been drastically restricted by the costly and time-consuming process of discovering biologically active compounds. Computer-aided drug design (CADD) provides an effective and relatively inexpensive method of identifying lead compounds [1]. Structure-based molecular docking, with conformational sampling and assessment of binding affinity, is a key element of CADD [2]. Improving the docking accuracy is paramount for enhancing success rates during virtual screening when undertaking computational drug development. Due to recent advances in computing power and numerical algorithms, docking success is no longer restricted by the inadequacies of conformational sampling [2, 3]. Most well-known docking software programs such as GOLD [4], AutoDockVina [5], and Glide [6], demonstrate excellent conformational sampling performance. However, there is presently a lack of accurate and reliable scoring functions available to evaluate the binding free energy between proteins and ligands, limiting the success rates of virtual screening within the drug discovery pipeline [2].

The scoring function is a mathematical model used to estimate the free energy of protein–ligand complexes, and helps to predict their binding affinities. It can be used to determine the binding mode of a ligand, predict the binding affinity between proteins and ligands, and identify the potential lead compounds for a given drug target. A precise and reliable scoring function is therefore critical for the success of any docking method or docking software [7]. Despite its importance, developing a precise and reliable scoring function is very challenging because the binding free energy between a protein and its ligand is very complex. Features such as van der Waals interactions, electrostatic interactions, hydrogen bonds, hydrophobic interactions, solvent effects, and the difficulty in capturing entropic contributions add to the complexity of this task [2]. Hence, the research into developing a more accurate and reliable scoring function is always a hot topic as it plays such an important role in computational drug development.

Conventional methods for scoring functions are usually classified into physics-based, empirical, and knowledge-based methods. In recent years, another category of scoring function based on machine learning (ML) has emerged as a fast yet accurate binding affinity prediction method [8–13]. Scoring power refers to the ability of a scoring function to produce binding scores in a linear correlation with experimental binding data [14]. The ML-based predictors usually perform better in the ‘scoring power’ of scoring functions than conventional methods. Early examples such as RFscores [8] and NNScore [9], which were based on random forest and neural network respectively, both applied ML methods to produce binding affinity predictions. These two scoring function models also relied on experts to perform very complex feature extractions. Later, deep convolutional neural network (CNN) models were adopted to undertake binding affinity predictions and virtual screening [13, 15–18]. AtomNet [18] is the first CNN model to predict the bioactivity of small molecules. K_DEEP_ [10] and Pafnucy [12] were also based on the CNN model, and both took the vectorized grids within a cubic box centered at the ligand as the features for the protein−ligand complex. Both K_DEEP_ and Pafnucy performed much better in terms of scoring power than the scoring functions based on conventional methods. Gnina is a deep learning framework for molecular docking [17, 19]. Gnina was trained by integrating non-binding data, and performed well on pose selection and affinity prediction. In addition, other features such as the protein–ligand topological fingerprints were also adopted for ML and CNN models [13, 20].

In this study, a much more concise method of featurization for the protein–ligand complex was adopted, generating a new scoring function model to predict binding affinities between proteins and their ligands after receiving training from a deep three-dimensional (3D) convolutional neural network. The features for the protein−ligand complex in our model were represented using a 3D grid or 4D tensor. In contrast to K_DEEP_ and Pafnucy models, the featurization of atoms or voxels in our model was simplified, and only the most basic atomic type information was extracted. The high dimensional information from protein–ligand complexes, including van der Waals interactions, electrostatic interactions, hydrogen bonds, and other complicating factors, were automatically learned by the subsequent convolutional neural network. Our scoring function model achieved a root mean squared error (RMSE) of 1.3263 and 1.4518 on CASF-2016 [14] and CASF-2013 datasets [21], respectively. Consistently, corresponding Pearson correlation coefficient R values of 0.7928 and 0.7946 were achieved by our model on these two datasets. Additionally, some independent extra sets were also selected to further evaluate the new model. As a result, compared to some other scoring functions, our model performed well, and was more stable in terms of scoring power. The model was implemented with TensorFlow [22] and Keras. The source code, trained model, and preprocessing scripts are available in the git repository at https://github.com/bioinfocqupt/Sfcnn.

## Methods

### Datasets

The scoring function model was trained with protein–ligand complexes from the refined set of the PDBbind database version 2019 [23]. This dataset contains 4852 high-quality protein−ligand complexes and their corresponding binding affinities expressed with p*K*_a_ (-lg*K*_*d*_ or -lg*K*_*i*_) values. Firstly, the CASF-2016 ‘scoring power’ benchmark [14] was selected as the test set. There were 285 protein–ligand complexes within the test set. All of the overlaps between the test set and the refined set were excluded from the refined set (266 overlaps). Then, for the remaining 4586 complexes in the v2016 refined set, 486 complexes (~ 10%) were randomly selected and used as the validation set. Finally, the remaining 4100 complexes (~ 90%) were adopted for the training set.

The CASF-2013 ‘scoring power’ benchmark [21], a subset of the PDBbind database version 2013, was selected as an extra test set in order to further compare the performance of our model with other scoring functions. The overlapping complexes which existed in the training and validation sets were removed from the CASF-2013 dataset. The remaining 107 complexes (referred to as the CASF-2013 dataset hereafter) were found to be a subset of the CASF-2016 dataset (the first test set).

Other independent test sets including CSAR_HiQ_NRC_set [24] (343 protein–ligand complexes), and Astex_diverse_set [25] (74 protein–ligand complexes), were also selected as extra test sets with the purpose of comparing the performance of our model with other scoring functions more fairly.

### Featurization of protein–ligand complexes

**Fig. 1:**
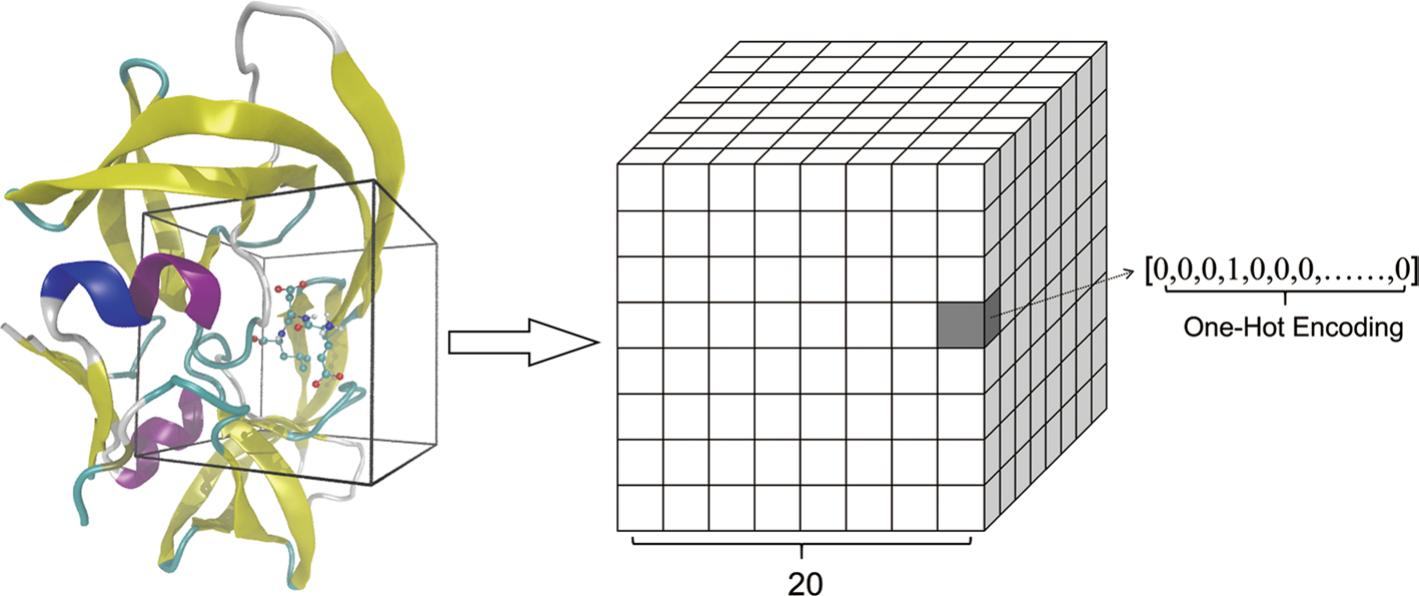
Featurization of the protein–ligand complexes. PDB ID 1a30 is shown as an example. In the default case, the resolution of 20 × 20 × 20 and 28 categories of atomic types were used

In our model, the protein–ligand complexes were transformed into a 3D grid for subsequent CNN training (Fig. 1). Firstly, the geometric center of the binding site was calculated by the coordinates of the atoms in the ligand. Then, a cube of 20 × 20 × 20 Å^3^ was cropped around the center of the binding site. All of the atoms (including protein atoms and ligand atoms, together with water, metal, and hydrogen atoms in the protein–ligand complex) within this cube area were retained for training. In the default case, the input 3D grid had a resolution of 20 × 20 × 20, and each voxel represented the atoms inside that 1 × 1 × 1 Å^3^ area. Finally, the 3D grid was further transformed into a 4D tensor. The first 3 dimensions of the 4D tensor represented the index of a voxel in the 3D grid, and the last dimension of the 4D tensor was a vector of features encoded by the atoms inside that voxel. For example, when given a 3D grid space between (− 10 Å, − 10 Å, − 10 Å) and (10 Å, 10 Å, 10 Å) with the origin at the geometric center of the binding site, and a carbon atom with coordinates of (− 0.5 Å, − 3.2 Å, 0.3 Å), it was assumed that the 4D tensor was represented by ***T***, and the vector of features encoded by this carton atom was represented by ***V***. Subsequently, the index of this atom was calculated in the 3D grid (which was (6, 9, 10)) and the corresponding 4D tensor of the voxel containing this atom could be represented as ***T*** (6, 9, 10) = ***V***. Each atom in the cube would be calculated in this manner and finally the protein–ligand complex would be transformed into a 4D tensor for subsequent CNN training. When multiple atoms were present in a single voxel, features from all of the atoms would be added.

As highlighted above, the last dimension of the 4D tensor is a vector of features encoded by the atoms in the small cube (1 × 1 × 1 Å^3^) area, and the vector needs to distinguish and differentiate all kinds of atomic types. Unlike the methods by which features were extracted in K_DEEP_ and Pafnucy, we simplified the featurization and only used one-hot encoding of atomic types as the input vectors of features. To better distinguish various atomic types, and to get better results of featurization, the atoms within protein–ligand complexes were classified into 28 categories, with 14 categories for proteins and 14 categories for ligands as the default setting (Additional file 1: Table S1).

The method used for protein–ligand complex featurization used in the default case, as described above, was named Feature1. A higher resolution of 24 × 24 × 24 was also considered when generating a 3D grid to test whether the resolution of 20 × 20 × 20 used in the default case could retain enough information to precisely predict the binding free energy of proteins and ligands. The method of featurization when using a resolution of 24 × 24 × 24, and retaining all atoms including hydrogen atoms and metal atoms for one-hot encoding (28 categories of atomic types just like used in the default case), was named Feature2. Taking into account that in many scoring functions, the water molecules and ions were removed and the hydrogen atoms and metal atoms were ignored prior to protein–ligand complex featurization, the third method of featurization was also adopted and named Feature3. This method used a resolution of 20 × 20 × 20 and ignored all of the hydrogen atoms and metal atoms when one-hot encoding of atomic types for comparison (24 categories of atomic types). The atomic features were calculated using Open Babel [26], and the script used for transforming the protein–ligand complex into a 4D tensor is also available at https://github.com/bioinfocqupt/Sfcnn.

### Network

During this work, the deep 3D convolutional neural network was used with a single output neuron to predict the binding affinity. To improve the performance of the scoring function, data enhancement was performed on the training set by randomly rotating each protein–ligand complex 9 times. The final training set consisted of 41,000 samples. All labels (the p*K*_a_ values of corresponding protein–ligand complexes) in the training and validation sets were normalized to range between zero and one by dividing each value by 15, thereby facilitating subsequent training. The Keras package with TensorFlow was used to construct the deep neural network architectures.

**Fig. 2:**
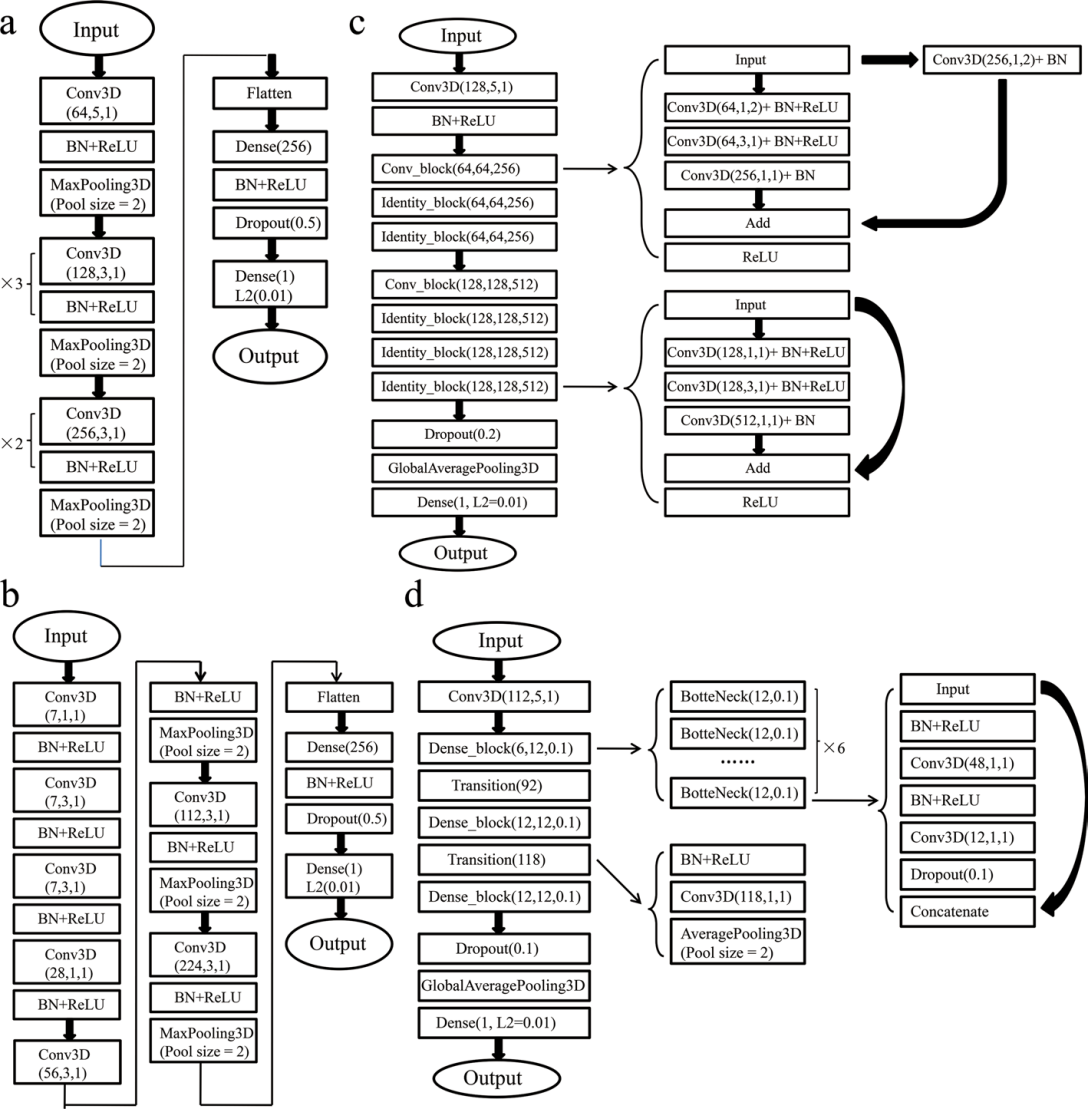
Architectures specifics. **a** Details of the CNN1 architecture. **b** Details of the CNN2 architecture. **c** Details of the Res3 architecture based on ResNet. **d** Details of the Dense4 architecture based on DenseNet. Abbreviations are defined as: Conv3D (number of channels, kernel size, stride): 3D convolutional neural network layer, BN: Batch Normalization layer, ReLU: Rectified Linear Unit activation layer

A total of 4 architectures were adopted for comparison during this work. The first architecture (called CNN1 hereafter) is a commonly used CNN architecture (Fig. 2a). This architecture takes the 4D tensor of a protein–ligand complex as the input. The first several convolutional layers extract features among nearby atoms. The following fully connected layers reorganize the features and predict the binding affinities between the proteins and ligands. ReLU activation and batch normalization were applied on each convolutional layer and fully connected layer. A dropout layer was applied after the fully connected layer and L2 regularization was applied on the output layer to reduce the probability of overfitting and improve generalization. The second architecture (called CNN2 hereafter) starts with a convolutional layer with a 1 × 1 × 1 filter (Fig. 2b). Because the input features in the present work were very sparse, using the convolutional layer with a 1 × 1 × 1 filter as the first layer enables mapping of the sparse feature vectors of the atoms to dense vectors and works like word embedding. This method may improve the performance. The other details of the CNN2 architecture are similar to the CNN1 as shown in Fig. 2b. The third architecture (called Res3 hereafter) is based on Resnet [27]. Resnet is a classical deep CNN architecture and gets outstanding performance in image recognition by training a deeper neural network with shortcut connection. In the present work, the Resnet architecture was transplanted to our 3D CNN training task. The detailed architecture of Res3 is shown in Fig. 2c. The fourth architecture (called Dense4 hereafter) is based on Densenet [28]. Densenet is another classical deep CNN architecture with fewer parameters and also has outstanding performance in image recognition. In the present work, the Dense architecture was transplanted to our 3D CNN training task and the detailed architecture is shown in Fig. 2d. Both Res3 and Dense4 used much deeper neural networks, requiring larger computational resources and longer computational time during training. The hyper-parameters of these four architectures including learning rate, batch size, dropout ratio, and L2 weight value were optimized by using the grid search method. Only the model with the lowest loss on the validation set for each architecture was saved for comparison.

### Evaluation

Several evaluation metrics were used to assess the model accuracy including root mean squared error (RMSE, which quantifies the relative deviations of the predicted values from the true values), mean absolute error (MAE, the average of the summed absolute differences of the predicted values to the true values), standard deviation (SD) and Pearson correlation coefficient (R) between the predicted p*K*_*a*_ (represented by *y*_*predict*_) and the experimentally determined true p*K*_*a*_ (represented by *y*_*true*_) in this study. The last two evaluation metrics were also adopted in the CASF-2016 benchmark dataset, therefore the accuracies of these scoring functions in the CASF-2016 benchmark were recalculated for comparison. The formulae for calculating the metrics of RMSE, MAE, SD, and R were as follows.

1 $${\text{RMSE}} = \sqrt {\frac{1}{N}\mathop \sum \limits_{i = 1}^{N} \left( {y_{predict} - y_{true} } \right)}$$

2 $${\text{MAE}} = \frac{1}{N}\sum \left| {y_{predict} - y_{true} } \right|$$

3 $${\text{ SD}} = \sqrt {\frac{1}{N - 1}\mathop \sum \limits_{i = 1}^{N} \left( {\left( {a*y_{predict} + b} \right) - y_{true} } \right)^{2} }$$

4 $${\text{R}} = \frac{{E\left( {y_{predict} *y_{true} } \right) - E\left( {y_{predict} } \right)*E\left( {y_{true} } \right)}}{{\sigma_{{y_{predict} }} \sigma_{{y_{true} }} }}$$

where a and b represent the slope and interception of the linear regression line of the predicted and measured p*K*_*a*_ values.

The DUD-E benchmark [29] was used to assess virtual screening abilities of Sfcnn. DUD-E benchmark consists of 102 targets, a set of active compounds known to bind these targets, and a lot of decoys for each active one. The DUD-E is a huge dataset consisting of over one million compounds and every target has a different number of active compounds to bind it. In this study we only selected a subset of DUD-E benchmark by random sampling to reduce computational time for docking and to ensure that every target corresponds to the same number of active compounds as possible. The fgfr1 (missing decoy data) and ace (containing silicon atoms) targets were excluded first. Then, we randomly selected 20 active compounds and 1000 decoys for each target to ensure the ratio of the number of active compounds to the number of decoys was 1:50. Finally, we performed molecular docking using Smina [5, 30] with default setting and re-scored the top 3 poses with Sfcnn. The 5% and 0.5% enrichment factors (EF) were used to assess the virtual screening ability of Sfcnn on each target. Also, we further tested the virtual screening ability of Sfcnn using a similar virtual screening approach on the CASF-2016 benchmark.

## Results and discussion

### Performance comparisons on four architectures with three methods of featurization

Table 1 demonstrates the best performance on the validation set after parameter tuning on the four different architectures using three methods of featurization as input. From Table 1, the CNN2 architecture, which begins with a convolution layer with a 1 × 1 × 1 filter, had significantly better performance than the other architectures when using Feature1 (resolution of 20 × 20 × 20, with all atoms retained) as input.

**Table 1:** The best performance model by training four architectures with three featurization methods as input. Feature1 used a resolution of 20 × 20 × 20 and retained all atoms. Feature2 used a resolution of 24 × 24 × 24 and also retained all atoms. Feature3 used a resolution of 20 × 20 × 20 but ignored hydrogen and metal atoms

Architecture	Feature1	Feature2	Feature3
CNN1	0.0099	0.0104	0.0100
CNN2	**0.0083**	0.0089	0.0095
Res3	0.0092	0.0102	0.0103
Dense4	0.0101	0.0104	0.0101

The best performance model on the validation set selected as the new scoring function model and named Sfcnn

Comparing with the architectures using Feature2, which adopted a higher resolution of 24 × 24 × 24 as input, the architectures using Feature1 with a resolution of 20 × 20 × 20 as input showed better performance in every architecture, thus indicating that a higher resolution or larger grid cannot significantly improve abilities relating to predicting the affinities between proteins and ligands. A higher resolution usually denotes that the input grid keeps more information from the protein–ligand complex but also represents that it needs increased computing resources to optimize the model. It is well known that the binding free energy between a protein and a ligand is contributed to by van der Waals interactions, electrostatic interactions, hydrogen bonds, hydrophobic interactions, solvent effects, and entropic contributions. Among the interactions between a protein and a ligand, the van der Waals interactions, hydrogen bonds, hydrophobic interactions, solvent effects, and entropic contributions are mainly localized around the ligand, and the 20 × 20 × 20 grid is good enough to collect these interactions. The electrostatic interactions, which are also very important in protein–ligand interactions, are long-range interactions and may not be fully accounted for within the 20 × 20 × 20 grid [13, 31]. However, as shown by the results in Table 1, generating the input tensors with resolution of the 20 × 20 × 20 is high enough for our prediction model. We presume that a larger grid may retain more noise by using our featurization methods of complexes. Smaller grids were also not considered because they do not collect most interactions between proteins and ligands sufficiently.

Comparisons of the architectures using Feature3, which ignores the hydrogen atoms and metal atoms in the protein–ligand complexes as input, against the architectures using Feature1 in which all of the atoms were retained, showed that the latter generally performed better. It is widely known that some hydrogen and metal atoms play very important roles in the interactions between proteins and their ligands, therefore retaining the hydrogen and metal atoms within the featurization of complexes is necessary to improve prediction abilities. This is also confirmed by the results in Table 1.

Comparisons between the CNN1, Res3, and Dense4 architectures showed that the CNN2 architecture generally demonstrated better performance levels. In our work, the deeper architecture based on classical Resnet and Densenet did not improve upon this performance. This may be related to the featurization method conducted in this study. Meanwhile, the Res3 and Dense4 architectures need increased levels of computing resources to handle the training, suggesting that they are not good architectures for our scoring function study. Because the input features in our work were very sparse, using the convolution layer with a 1 × 1 × 1 filter as the first layer enables mapping of the sparse feature vectors of the atoms to dense vectors and works in a similar manner to word embedding. We suspect this may be the reason that the CNN2 architecture exhibited the best performance.

The best performance model generated by the CNN2 architecture with Feature1 as input was chosen as the new scoring function model, named Sfcnn, for subsequent analysis. The total number of parameters for this model was 1,354,588. When training was undertaken for this model, the RMSprop optimizer was used with a 0.004 learning rate and 64 batch sizes. Other optimizers, learning rates, and batch sizes were tested but resulted in worse performance. To reduce overfitting, the dropout approach was used in the full connect layer with a 0.5 drop rate and L2 weight decay in the last layer with 0.01. Other values were also tested and resulted in higher losses. The best model was obtained with a minimal loss for the validating set at epoch = 112 (Additional file 1: Fig. S1).

### Sfcnn performance on the training, validation, and test sets

**Fig. 3:**
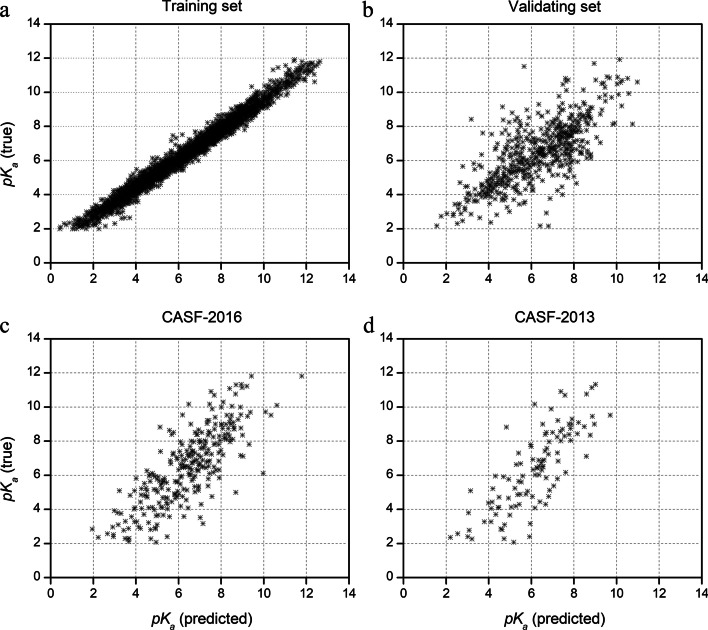
Performance of the Sfcnn model on two test sets (CASF-2016 and CASF-2013 datasets), training set, and validation set

The prediction accuracy of the Sfcnn model was determined based on the following evaluation metrics: RMSE, SD, MAE, and R. The Sfcnn model’s performance on the training, validation, and test sets are shown in Table 2. R = 0.9894 was achieved on the training set whereas R = 0.7336 on the validation set. By evaluating the performance on the CASF-2016 test set, the Sfcnn model achieved R (0.7928) close to 0.8 and a relatively small RMSE (1.3263). The performance on the CASF-2016 test set was slightly less than that of OnionNet [13] (R = 0.816) and AGL [32] (R = 0.833) which are also based on deep learning as previously reported by Zheng et al. [13]. However, when they were evaluated on the CASF-2013 test set, the performance of our Sfcnn model was slightly better than that of OnionNet (R = 0.78) and AGL (R = 0.792) [13], and also achieved an R (0.7946) close to 0.8 with a relatively small RMSE (1.4518). Overall, the performance of Sfcnn was similar to that of Onion and AGL, and all of them achieved a pretty good performance level for scoring power with an R around 0.8 and a relatively small RMSE. Meanwhile, the predicted p*K*_a_ and the true p*K*_a_ were highly linear correlated for the two test sets and the validating set as shown in Fig. 3.

**Table 2:** Performance of Sfcnn on training, validation, CASF-2016, and CASF-2013 datasets

Dataset	R	RMSE	MAE	SD
Training set	0.9894	0.4402	0.3474	0.2854
Validation set	0.7336	1.2981	0.9391	1.2159
CASF-2016	0.7928	1.3263	1.0277	1.3253
CASF-2013	0.7946	1.4518	1.1139	1.4165

### Comparison with other scoring functions evaluated on the CASF-2016 benchmark

Sfcnn and other scoring functions were also compared in terms of their abilities relating to scoring powers for predicting protein–ligand binding affinities using the CASF-2016 dataset. Table 3 shows the R and SD values given by Sfcnn and the top 10 scoring functions tested on the CASF-2016 benchmark by Su et al. [14]. As shown in Table 3, the Sfcnn model ranked 2nd among the 11 scoring functions. Among the top 10 scoring functions of CASF-2016, only ΔvinaRF_20_ [33] is based on machine learning, while the others could all be classified into conventional scoring functions based on physics, empirical, or knowledge. The best performing X-score in conventional scoring functions achieves R = 0.631 and SD = 1.69, while our Sfcnn model achieved R = 0.792 and SD = 1.32, indicating that Sfcnn performed significantly better than all conventional scoring functions for scoring power. However, the Sfcnn model was still a little worse than ΔvinaRF_20_ which achieved R = 0.816 and SD = 1.26 for scoring power. The performances of Sfcnn and ΔvinaRF_20_ were further compared on the CASF-2013 benchmark [21]. ΔvinaRF_20_ only achieved R = 0.646 on the CASF-2013 dataset as reported by Wang et al. [33]. This performance was significantly worse than that of Sfcnn, which achieved R = 0.7946 on CASF-2013 (Table 3).

**Table 3:** Comparison between Sfcnn and the top 10 scoring functions tested on the CASF-2016 benchmark

Scoring function	R	SD	Size	Description
ΔVinaRF_20_	0.816	1.26	285	Machine learning
Sfcnn	0.792	1.32	283	Machine learning
X-Score	0.631	1.69	285	Empirical
X-ScoreHS	0.629	1.69	285	Empirical
ΔSAS	0.625	1.7	285	Single descriptor
X-ScoreHP	0.621	1.7	285	Empirical
ASP@GOLD	0.617	1.71	282	Knowledge-based
ChemPLP@GOLD	0.614	1.72	281	Empirical
X-ScoreHM	0.609	1.73	285	Empirical
AutoDockVina	0.604	1.73	285	Empirical
DrugScore2018	0.602	1.74	285	Knowledge-based

Results (excluding Sfcnn) cited from Su et al. [14]. The performance of these scoring functions was recalculated by us for comparison

Why were the performances of Sfcnn and ΔvinaRF_20_ different when using the CASF-2016 and CASF-2013 datasets? We suspect that there may be some overlaps between the CASF-2016 test set and the training set used for ΔvinaRF_20_. As reported by Wang et al. [33], the main training set of ΔvinaRF_20_ was the v2014 refined set [34] from the PDBbind database, after removal of the overlaps with the CASF-2007 [35] and CASF-2013 [21] datasets, which were the test sets used in the study of ΔvinaRF_20_. We collected the v2014 refined set, CASF-2007 dataset, and CASF-2013 dataset from the PDBbind database. Then any structure in the CASF-2007 and CASF-2013 datasets was excluded from the v2014 refined set to get the main training set of ΔvinaRF_20_ named refined-2014*. There were 140 complexes present in both the refined-2014* and the CASF-2016 dataset (Additional file 1: Fig. S2), suggesting that the performance of ΔvinaRF_20_ on the CASF-2016 dataset was falsely high. According to the performances observed for Sfcnn and ΔvinaRF_20_ on the CASF-2013 dataset, the scoring power of Sfcnn was still better than that of ΔvinaRF_20_, and the Sfcnn model demonstrated a highly excellent performance when predicting the binding ability of proteins and ligands.

### Evaluating the performance on CASF-2013, CSAR_HiQ_NRC_set and Astex_diverse_set

To further, and fairly, evaluate the performance of Sfcnn, some additional scoring functions were applied including DeepBindRG, AutoDockVina, and Pafnucy for comparison. Both DeepBindRG and Pafnucy were based on deep learning and showed excellent performance with regards to scoring power. AutoDockVina is a very popular docking software, and its scoring function is based on empirical. Additionally, some extra independent test datasets were tested including the CASF-2013 dataset, the Astex_diverse_set, and the CASR_HiQ_NRC_set to evaluate the scoring power of Sfcnn, DeepBindRG, AutoDockVina, and Pafnucy. The performance of these scoring functions on the three extra test datasets is presented in Table 4, using R, RMSE, and MAE values as performance indicators.

**Table 4:** Performance of Sfcnn, DeepBindRG, AutoDockVina, and Pafnucy on CASF-2013, CSAR_HiQ_NRC_set, and Astex_diverse_set datasets

Dataset	R	RMSE	MAE	Size
Sfcnn performance				
CASF-2013	0.7946	1.4518	1.1139	107
CSAR_HiQ_NRC_set	0.824	1.277	0.8375	343
CSAR_HiQ_NRC_set*	0.6758	1.8079	1.3680	149
Astex_diverse_set	0.6474	1.3627	1.0518	74
DeepBindRG performance				
CASF-2103	0.6394	1.817	1.4829	195
CSAR_HiQ_NRC_set	0.6585	1.7239	1.3607	343
Astex_diverse_set	0.4657	1.6209	1.3355	74
AutoDockVina performance				
CASF-2103	0.5725	2.401	1.9462	195
CSAR_HiQ_NRC_set	0.5707	2.2884	1.7268	343
Astex_diverse_set	0.422	2.2027	1.7068	74
Pafnucy performance				
CASF-2103	0.5855	1.8491	1.5131	195
CSAR_HiQ_NRC_set	0.6693	1.6805	1.3336	343
CSAR_HiQ_NRC_set*	0.7040	1.8868	1.5230	136
Astex_diverse_set	0.5146	1.4654	1.1732	74

Results (excluding all the Sfcnn performance and the Pafnucy performance on CSAR_HiQ_NRC_set* dataset) cited from Zhang et al. [36]

*indicates the dataset after removal of the overlaps

For the performance of Pafnucy on CASF-2013, Stepniewska-Dziubinska et al. [12] reported that the R value of Pafnucy achieved R = 0.70, while Zhang et al. [36] reported it only achieved R = 0.5885. We have recalculated the performance of Pafnucy on the CASF-2013 dataset and found it was closer to the latter (R = 0.544 by our calculations), therefore we chose the Pafnucy performance tested by Zhang et al. [36] for comparison. As shown in Table 4, the performance of Sfcnn on CASF-2013 achieved R = 0.7946 and RMSE = 1.4518, and this performance was significantly better than that of the others.

On the full CSAR_HiQ_NRC_set, the R value of Sfcnn achieved 0.824. However, there were 194 complexes present in both the CSAR_HiQ_NRC_set and the training set for Sfcnn. Therefore, the Sfcnn performances were recalculated on the CSAR_HiQ_NRC_set after removal of the overlaps (represented by CSAR_HiQ_NRC_set* in Table 4), and the R value of Sfcnn decreased to 0.6758, which was still better than that of DeepBindRG and AutoDockVina. To assess the performance of Pafnucy on the CSAR_HiQ_NRC_set, the results calculated by us were adopted because there were also many overlaps between the CSAR_HiQ_NRC_set and the Pafnucy training set, and the study by Zhang et al. [36] did not give the real performance levels of Pafnucy following removal of the overlaps from the CSAR_HiQ_NRC_set. As shown in Table 4, the R value of Pafnucy was 0.6693 but achieved 0.7040 after the overlaps were removed. This performance was a little better than that of the Sfcnn model. However, both the RMSE and MAE values of Pafnucy were higher than that of Sfcnn, suggesting that Sfcnn’s ability to predict the binding affinity was more stable. In general, DeepBindRG, Pafnucy, and Sfcnn displayed similar performances on the CSAR_HiQ_NRC_set, and they all showed better scoring powers than AutoDockVina. For the Astex_diverse_set, the performance of Sfcnn was significantly better than the others for the R, RMSE, and MAE values. Overall, the Sfcnn model performed well for two of three datasets and showed an excellent performance regarding predicting the binding affinity between a protein and ligand.

Taking into account that high structural and chemical similarity of the protein and ligand between training set complexes and test ones may overestimate the performance of scoring functions [37, 38], we further performed similarity test between training and test sets. Protein structural similarity was computed by TM-Score and ligand similarity was computed by RDkit’s [17, 39, 40]. Complexes with TM-Score of over 0.5 or 0.17 and ligand similarity of over 0.8 to the ones in training set were excluded from the test sets. As shown in Table 5, after excluding complexes with high structural and chemical similarity, the performance of Sfcnn still perform well on all test sets. Sfcnn still achieves an R value of over 0.77 on CASF-2016 and CASF-2013 datasets, maintaining a good performance. On CSAR_HiQ_NRC_set and Astex_diverse_set, the R value of Sfcnn still achieves an R value of over 0.6. Overall, after excluding complexes with high structural and chemical similarity, Sfcnn still maintains a good performance on all test sets, which may be related to the featurization method we have adopted. As in our featurization method, the input 4D tensor features will not be the same as long as the coordinates of the complexes are not identical.

**Table 5:** Performance of Sfcnn on CASF-2016, CASF-2013, CSAR_HiQ_NRC_set, and Astex_diverse_set datasets after excluding complexes with high structural and chemical similarity to the training set ones

Datasets	R	RMSE	MAE	Size
TM < 0.5, ligand similarity < 0.8				
CASF-2016	0.7772	1.4006	1.0931	200
CASF-2013	0.7898	1.5592	1.1882	78
CSAR_HiQ_NRC_set	0.6372	1.8839	1.4630	124
Astex_diverse_set	0.6404	1.3372	1.0505	70
TM < 0.17, ligand similarity < 0.8				
CASF-2016	0.8008	1.3731	1.087	170
CASF-2013	0.7863	1.6088	1.2282	65
CSAR_HiQ_NRC_set	0.6353	1.9321	1.5149	75
Astex_diverse_set	0.6356	1.3109	1.0425	53

### Virtual screening performance on the CASF and DUD-E benchmarks

**Fig. 4:**
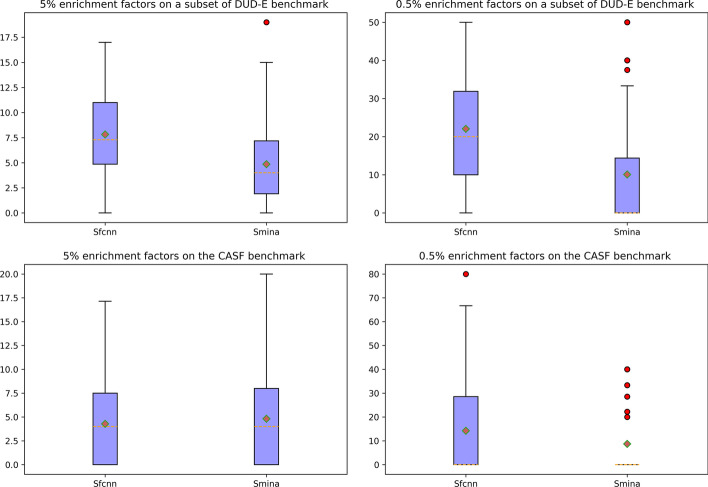
Virtual screening performance of Sfcnn and Smina on CASF-2016 benchmark and a subset of DUD-E benchmark

Docking power refers to the ability of a scoring function to identify the native ligand binding pose among computer generated decoys. We further tested the docking power of Sfcnn on CASF-2016 benchmark and assessed the ability of Sfcnn to identify those poses with a RMSD within 2 Å from the native one. The top one, top two and top three predictions of Sfcnn are 34%, 50.2% and 58.9% on the CASF-2016 benchmarks, respectively. This performance is far lower than the docking power of AutoDockVina, which is the best one tested by Su et al. [14], with a score of 90.2%, 95.8% and 97.2%. To the best of our knowledge, many ML-based scoring functions such as K_DEEP_, Pafnucy and AK-score [41] do not perform very well in terms of docking power, due to the lack of integration of non-binding data for training. But these scoring functions can be used in combination with AutoDockVina to re-score the pose identified by AutoDockVina to improve the success rate of virtual screening. In the virtual screening test of this study, we used Smina which is a fork of AutoDockVina to dock ligands to targets and then used Sfcnn to re-score these complexes. We first tested the virtual screening ability on the DUD-E benchmark. The DUD-E benchmark is a very popular and huge dataset with more than one million compounds, widely used for assessing virtual screening abilities of scoring functions and docking protocols. Due to computational resource limitations, we built a subset to evaluate the virtual screening ability of Sfcnn by randomly sampling the DUD-E dataset. In this subset, there were 20 active compounds and 1000 decoys for each target. We expected to identify the active ones from a total of 1020 small molecules by Sfcnn scoring function. As shown in Additional file 1: Table S2 and Fig. 4, the EF 5% and EF 0.5% for Sfcnn are significantly higher than that for Smina on this subset of DUD-E benchmark (Student’s test, *p*-value = 1.15e-06 and *p*-value = 9.74e-08, respectively), showing that Sfcnn has better virtual screening ability than Smina and AutoDockVina on this subset. However, there may be some decoys designed not against the actives on the subset, resulting in different distributions of actives and decoys on this subset and the whole DUD-E dataset. Therefore, the virtual screening result of Sfcnn in this study only illustrated its performance on a subset of DUD-E benchmark and this result may differ from the test result on the whole DUD-E benchmark. To remedy this deficiency, we further tested the virtual screening performance of Sfcnn on another smaller dataset. As shown in Additional file 1: Table S3 and Fig. 4, Sfcnn shows a virtual screening performance comparable to Smina On the CASF-2016 benchmark (Student’s test, *p*-value = 0.54 and *p*-value = 0.12, respectively). Overall, Sfcnn shows a good virtual screening performance.

### Explainable 3D CNN model using grad-CAM

Gradient-weighted class activation mapping (Grad-CAM) [42] is a widely used technique for making any CNN-based models more transparent. It produces visual explanations and helps determine more about the model when performing detection or prediction work. We utilized this method to identify the hot spot areas of the input feature that play important roles in determining the output predicted score. In this work, the last convolutional layer and the output predicted score of the full model were used when applying Grad-CAM. Then the heatmap of the last convolutional layer was resized to the same size with the input feature, and subsequently visualized using Mayavi [43].

**Fig. 5:**
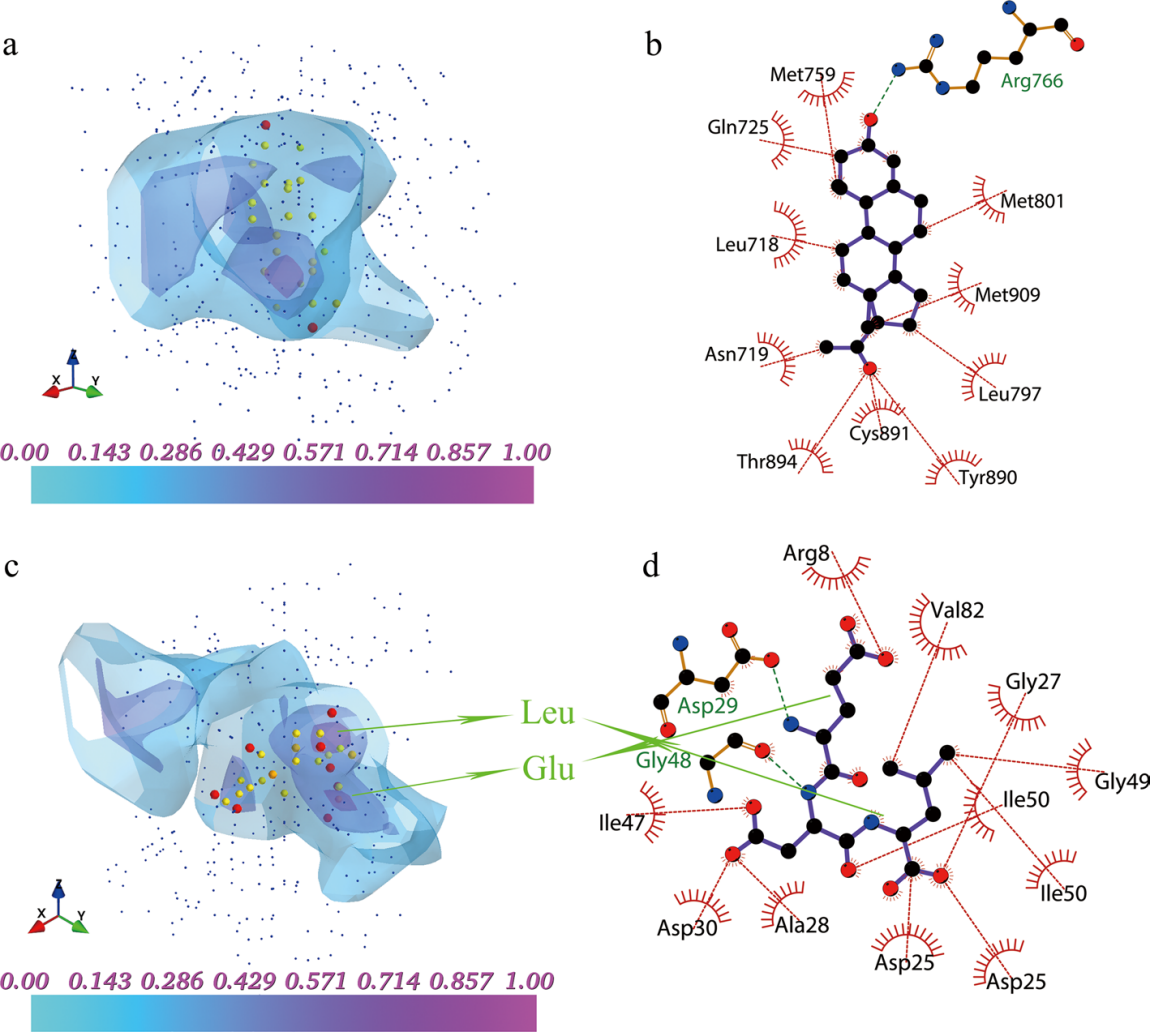
Heatmaps of Grad-CAM analyses and presentations of input features. **a** Example of a protein–ligand complex whose PDB ID was 1a28 from the training set. **b** 2D protein–ligand interactions of 1a28. **c** Example of a protein–ligand complex whose PDB ID was 1a30 from the CASF-2016 test set. **d** 2D protein–ligand interactions of 1a30. In **a** and **c**, the red spheres represent oxygen atoms of the ligand, the orange spheres represent nitrogen atoms of the ligand, the yellow spheres represent carbon atoms of the ligand and the small blue spheres represent the atoms of the protein. Hydrogen atoms are not shown on the graph for viewing purposes. The heatmap is colored from cyan to purple. In **b** and **d**, the green dashed line indicates hydrogen bonds and the red arc areas indicate hydrophobic interactions. **a** and **c** were drawn by Mayavi. **b** and **d** were generated with LigPlot + [46]

The example cases of Grad-CAM analysis on the protein–ligand complexes are illustrated in Fig. 5. Figure 5a and b show the structure of the hormone-bound human progesterone receptor complexed with progesterone from the training set (PDB ID: 1a28) [44]. As shown in Fig. 5a, the hot spot areas were mainly around the ligand, and the high activation area colored in purple was around the five-carbon ring of progesterone. This was consistent with the finding in Fig. 5b that there were strong hydrophobic interactions between the receptor and progesterone, suggesting that the hydrophobic interactions may play a dominant role in the binding of this protein to the ligand. Figure 5c and Fig. 5d depict the structure of HIV-1 protease complexed with a tripeptide inhibitor from the CASF-2016 test set (PDB ID: 1a30) [45]. As shown in Fig. 5c, the high activation areas colored in purple were around the Glu and Leu residues in the ligand. Meanwhile, the hydrogen bond had formed between ligand-Glu and protein-Asp29 and there were strong hydrophobic interactions between the protease and the Leu residue of the inhibitor, playing an important role in determining the binding affinity. Heatmaps of Grad-CAM analyses for the other convolutional layers are shown in Fig. S3 and Fig. S4. These examples showed that the features learned by convolutional layers are explainable, and they may represent the high dimensional information between the protein and ligand such as hydrophobic interactions and hydrogen bonds. Meanwhile, combining the CNN model with the Grad-CAM analysis may help identify the critical functional groups in determining the binding free energy between proteins and ligands.

## Conclusions

In the present work, we designed a convenient and reversible feature engineering method and developed a scoring function “Sfcnn” based on a deep 3D convolutional neural network, to improve ligand binding affinity prediction. Sfcnn enables a very easy, fast, and accurate calculation of the binding free energies between proteins and ligands. It is also capable of handling almost any docking result, and any ligand, regardless of the type of atoms in the ligand. Meanwhile, the feature engineering method used in this study enables a good reversible conversion between spatial structure and numerical features. The reversibility facilitates the intelligent design of novel drugs but has rarely considered on other scoring functions. Although the feature engineering in the Sfcnn model has been greatly simplified, Sfcnn still performs comparably to scoring functions such as OnionNet and AGL on the CASF-2016 and CASF-2013 datasets. The accuracy of Sfcnn was also comparable with all scoring functions provided by the CASF-2016 dataset, and Sfcnn showed the best performance for scoring power. Meanwhile, the Sfcnn model was also comparable with DeepBindRG and Pafnucy, which are both based on deep learning in several extra independent datasets. Sfcnn performed well on two of the three datasets tested. For the remaining dataset, Sfcnn also showed a performance comparable with DeepBindRG and Pafnucy. Overall, Sfcnn has shown a fairly stable and accurate prediction performance by evaluating Sfcnn and other scoring functions on different datasets. In addition, the visual high-level features automatically learned by convolutional layers provided interpretability for the superior performance of Sfcnn and this method can also be used to optimize the lead compound and find optimal pose during docking. These results indicate the Sfcnn model is an excellent scoring function, and performs well in scoring power for accurately and stably predicting the binding affinities between proteins and ligands. The Sfcnn model will contribute towards improving the success rate of virtual screening, thus will accelerate the development of potential drugs or novel biologically active lead compounds.

## Supplementary Information

**Additional file1:** **Table S1.** The atomic types used in this study. **Table S2.** 5% and 0.5% enrichment factors computed on the DUD-E benchmark for Sfcnn and Smina. **Table S3.** 5% and 0.5% enrichment factors computed on the CASF benchmark for Sfcnn and Smina. **Fig. S1.** The error of the Sfcnn model on training and validation sets during learning. **Fig. S2.** The overlaps between CASF-2016 and refined-2014* datasets. **Fig. S3.** Grad-CAM analyses of other convolutional layers for 1a28. The heatmap is colored from cyan to purple. The heatmap of Conv layer 3 does not contain positive activation area. **Fig. S4.** Grad-CAM analyses of other convolutional layers for 1a30. The heatmap is colored from cyan to purple.

## Data Availability

The source code, together with the preprocessing scripts, is available at https://github.com/bioinfocqupt/Sfcnn.

## Abbreviations

CADD: Computer-aided drug design
ML: Machine learning
CNN: Convolutional neural network
RMSE: Root mean squared error
MAE: Mean absolute error
SD: Standard deviation
Sfcnn: Scoring function based on deep 3D CNN
EF: Enrichment factors
