# Supplementary material for: Sfcnn: a novel scoring function based on 3D convolutional neural network for accurate and stable protein–ligand affinity prediction
Source: BMC Bioinformatics. 2022 Jun 8;23:222. doi: 10.1186/s12859-022-04762-3 (PMC9178885; doi:10.1186/s12859-022-04762-3)
Supplement: Supplementary file 1 — Additional file1: Table S1. The atomic types used in this study. Table S2. 5% and 0.5% enrichment factors computed on the DUD-E benchmark for Sfcnn and Smina. Table S3. 5% and 0.5% enrichment factors computed on the CASF benchmark for Sfcnn and Smina. Fig. S1. The error of the Sfcnn model on training and validation sets during learning. Fig. S2. The overlaps between CASF-2016 and refined-2014* datasets. Fig. S3. Grad-CAM analyses of other convolutional layers for 1a28. The heatmap is colored from cyan to purple. The heatmap of Conv layer 3 does not contain positive activation area. Fig. S4. Grad-CAM analyses of other convolutional layers for 1a30. The heatmap is colored from cyan to purple. [file 12859_2022_4762_MOESM1_ESM.docx]

**Additional file 1**

**Table S1:** The atomic types used in this study.

**Table S2:** 5% and 0.5% enrichment factors computed on the DUD-E benchmark for Sfcnn and Smina.

**Table S3:** 5% and 0.5% enrichment factors computed on the CASF benchmark for Sfcnn and Smina.

**Fig. S1:** The error of the Sfcnn model on training and validation sets during learning.

**Fig. S2:** The overlaps between CASF-2016 and refined-2014* datasets.

**Fig. S3:** Grad-CAM analyses of other convolutional layers for 1a28. The heatmap is colored from cyan to purple. The heatmap of Conv layer 3 does not contain positive activation area.

**Fig. S4:** Grad-CAM analyses of other convolutional layers for 1a30. The heatmap is colored from cyan to purple.
